# Antibacterial Activity of ZnSe, ZnSe-TiO_2_ and TiO_2_ Particles Tailored by Lysozyme Loading and Visible Light Irradiation

**DOI:** 10.3390/antiox12030691

**Published:** 2023-03-10

**Authors:** Crina Anastasescu, Simona Neagu, Silviu Preda, Daniela Culita, Mihaela Stancu, Cristian Banciu, Cornel Munteanu, Veronica Bratan, Jose Maria Calderon-Moreno, Razvan State, Mihai Anastasescu, Madalin Enache, Ioan Balint, Maria Zaharescu

**Affiliations:** 1 “Ilie Murgulescu” Institute of Physical Chemistry of the Romanian Academy, 202 Spl. Independentei, 060021 Bucharest, Romania; canastasescu@icf.ro (C.A.); predas@icf.ro (S.P.); danaculita@yahoo.co.uk (D.C.); munteanuc@icf.ro (C.M.); vbratan@yahoo.com (V.B.); calderon@icf.ro (J.M.C.-M.); rstate@icf.ro (R.S.); ibalint@icf.ro (I.B.); mzaharescu@icf.ro (M.Z.); 2 Institute of Biology of Romanian Academy, 296 Splaiul Independenţei, 060031 Bucharest, Romania; mihaela.stancu@ibiol.ro (M.S.); crisbanciu@gmail.com (C.B.); madalin.enache@ibiol.ro (M.E.)

**Keywords:** ZnSe, ZnSe-TiO_2_, TiO_2_, hybrid organic–inorganic, light absorption properties, reactive oxygen species generation, antibacterial activity in dark and visible light assisted

## Abstract

ZnSe, ZnSe-TiO_2_ microspheres and nanostructured TiO_2_ obtained by hydrothermal and sol–gel methods were tested against *Staphylococcus aureus* ATCC 25923 and *Micrococcus lysodeikticus* ATCC 4698 before and after lysozyme (Lys) loading. Morphological characterization of inorganic matrices and hybrid organic–inorganic complexes were performed by microscopy techniques (SEM, AFM and Dark Field Hyperspectral Microscopy). Light absorption properties of ZnSe, ZnSe-TiO_2_ and TiO_2_ powders were assessed by UV–visible spectroscopy and their ability to generate reactive oxygen species (•OH and O_2_^•−^) under visible light irradiation was investigated. Antibacterial activity of ZnSe, ZnSe-TiO_2_, TiO_2_, Lys/ZnSe, Lys/ZnSe-TiO_2_ and Lys/TiO_2_ samples under exposure to visible light irradiation (λ > 420 nm) was tested against *Staphylococcus aureus* and *Micrococcus lysodeikticus* and correlated with ROS photogeneration.

## 1. Introduction

In the last years, a large range of engineered materials have been studied in order to develop new antibacterial tools meant to support healthcare and life quality due to the excessive use of chemicals and antibiotics producing major imbalances in the beings’ behavior. Therefore, it is mandatory now to find new pathways able to limit the aggression of pathogens and their increasing resistance. For instance, the intensive use of biologically active compounds (antibacterial enzymes, antibodies and biological markers) [1,2] for biomedical application can be a useful approach since their drawbacks (high costs and short lifetime) are already known and can be overcome by embedding them in hybrid organic–inorganic systems with increased activity, reusability and higher endurance [3,4]. These should also contain valuable inorganic materials only for carrying the bioactive compounds or endow themselves with intrinsic antibacterial properties. The lysozyme (1,4-*N*-acetylmuramidase) is such a biologically active compound and an antibacterial enzyme present in the living world and human body causing cell wall lysis in Gram-positive bacteria. It is largely studied [5] both for its antibacterial and enzymatic properties [6,7,8] but also for its interactions in different media, such as model protein [9,10].

The bioactivity of synthetic inorganic materials was deeply investigated, with many studies focusing on SiO_2_ and TiO_2_ [11] obtained by the sol–gel method because it uses materials that are non-toxic, low cost and well-investigated, but many other inorganic structures, most of them targeting other application fields than biomaterials, are now subjected to the bioactivity assays, such as ZnSe-based materials [12,13]. ZnSe is an intrinsic semiconductor with a direct band gap of ~2.7 eV [14] used especially in optoelectronic and energy fields [15,16]. In our previous study, micro-spherical ZnSe, TiO_2_-ZnSe and their hybrid complexes with lysozyme were deeply characterized from a morphological, structural and functional point of view [17]. Enzymatic activity of hybrid materials was investigated relative to a synthetic substrate, namely 4-Methylumbelliferyl β-d-*N*,*N*′,*N*″-triacetylchitotrioside ([4-MU-β-(GlcNAc)_3_]), proving to be increased in comparison with the free enzyme. It is noteworthy to reveal their behavior toward a natural substrate, such as *Micrococcus lysodeikticus*, since there are studies that discriminate between the enzymatic and antibacterial activity of lysozymes [8,18].

Based on the above mentioned considerations, the present study aims to investigate the antibacterial activity of ZnSe, ZnSe-TiO_2_ and TiO_2_ materials before and after modification with lysozymes against *Staphylococcus aureus* and *Micrococcus lysodeikticus* and disclose their particularities but also to check the light sensitivity of ZnSe, ZnSe-TiO_2_ and TiO_2_ materials and their impact on the antibacterial activity against *Staphylococcus aureus* by means of reactive oxygen species (•OH and O_2_^•−^) generated under visible light exposure.

The main antibacterial mechanisms signaled by the literature are related to the generation of reactive oxygen species (hydroxyl radical, singlet oxygen, superoxide anion and hydrogen peroxide) [19,20]. Recent studies paid attention also to photogenerated ROS and the use of photoactive materials against pathogens, including bacterial biofilms and viruses [21,22,23].

Most of the above mentioned reports are centered on oxide materials, while the data concerning the ZnSe-based materials with antibacterial activity are fewer and, therefore, proving this study to novel and of high importance [24,25].

The aim of the present work is to test the antimicrobial activity of ZnSe, ZnSe-TiO_2_ and TiO_2_ materials against *Staphylococcus aureus* and *Micrococcus lysodeikticus* and to increase it by two procedures:

(a) Through formation of a hybrid complex by lysozyme loading (Lys/ZnSe, Lys/ZnSe-TiO_2_ and Lys/TiO_2_);
(b) By exposing the light sensitive samples (ZnSe, ZnSe-TiO_2_ and TiO_2_) and their hybrid complexes (Lys/ZnSe, Lys/ZnSe-TiO_2_ and Lys/TiO_2_) to visible light irradiation before the antibacterial assay on *Staphylococcus aureus* and *Micrococcus lysodeikticus*.

Photogeneration of reactive oxygen species (•OH and O_2_^•−^) over the samples of interest is envisaged in order to depict the particularities of the antibacterial mechanism for each sample. The effect of Lys/ZnSe-TiO_2_ on *Staphylococcus aureus* was also monitored by Dark field microscopy and the morphological particularities of lysozymes, *Staphylococcus aureus* and *Micrococcus lysodeikticus* before and after interaction with the investigated samples by atomic force microscopy.

## 2. Materials and Methods

### 2.1. Synthesis of Inorganic Matrices (ZnSe, ZnSe-TiO_2_, TiO_2_)

ZnSe and ZnSe-TiO_2_ microspheres were synthesized by the hydrothermal method according to our previous work [17] using sodium selenite (anhydrous Na_2_SeO_3_, 99% min, Alfa Aesar, Karlsruhe, Germany), zinc sulphate heptahydrate (ZnSO_4_·7H_2_O 99.5%, Carl Roth GMBH Karlsruhe, Germany), hydrazine monohydrate (N_2_H_4_·H_2_O 98%, Sigma Aldrich, St Luis, MO, USA) and NaOH (Alfa Aesar) precursors. The autoclave was kept at 140 °C for 3 h. In order to obtain the ZnSe-TiO_2_ composite, TiO_2_ nanoparticles were previously synthesized by sol–gel method starting from titanium isopropoxide (97%, Aldrich) and 2, 4-Pentanedione (Alfa Aesar) [26], dried and calcined at 400 °C for one hour. Subsequently, TiO_2_ nanoparticles were added to the same mixture, as in the case of ZnSe synthesis, and then introduced in an autoclave for the hydrothermal treatment. 

For a valid comparison between the investigated materials and their properties, the TiO_2_ sample studied in this work was subjected after sol–gel synthesis to the same hydrothermal treatment (aqueous hydrazine and NaOH media) as in the case of ZnSe and ZnSe-TiO_2_ samples.

### 2.2. Lysozyme Adsorption on ZnSe, ZnSe-TiO_2_ and TiO_2_ Samples

Lysozymes (obtained from chicken egg white) and potassium phosphate buffer solution (PBS, pH 6.5) were provided by Lysozyme Activity Kit—LY0100 Sigma Aldrich (Sigma, Product of USA, St Luis). A total of 0.01 g of ZnSe, ZnSe-TiO_2_ and TiO_2_ powders were dispersed in 10 mL PBS containing lysozyme (0.25 mg/mL) and then gently shaken for 4 h at 25 °C. The solid samples were recovered by centrifugation (10,000 rpm for 10 min), washed with PBS/ultrapure water and dried at vacuum for further investigation, such as AFM and antibacterial characterization. Supernatants containing lysozyme were characterized by UV–visible spectroscopy. Additionally, the PBS containing lysozyme sand *Micrococcus lysodeikticus* ATCC 4698 0.01% *w/v* (Lysozyme Activity Kit—LY0100 Sigma Aldrich) was used for preparation of AFM samples by drop-casting on Si or glass substrates.

### 2.3. Characterization Methods

#### 2.3.1. Scanning Electron Microscopy (SEM)

SEM images have been obtained using a high-resolution microscope, Quanta 3D (FEI, Eindhoven, The Netherlands) at operating voltages between 5 and 20 kV.

#### 2.3.2. Dark field microscopy

Dark field hyperspectral microscopy characterization was carried out using a Cyto Viva Hyperspectral Microscope in order to capture optical and hyperspectral images of *Staphylococcus aureus* in the presence of the lysozyme/ZnSe-TiO_2_ complex. In this sense, *S. aureus* cell suspension (prepared according to Antibacterial assay section) was exposed for 4 h to lysozyme/ZnSe-TiO_2_ and maintained at 37 °C by using a shaker incubator. A droplet of the sample was applied and left to dry on a microscope slide.

#### 2.3.3. Atomic force microscopy (AFM)

Atomic force microscopy (AFM) measurements were made in non-contact mode with XE-100 from Park Systems. XE100 (Park Systems, Suwon, Republic of Korea) microscope used flexure-guided, crosstalk eliminated scanners thus minimizing the tip-sample interaction. All AFM images were recorded with sharp tips, NCHR-type (Nanosensors^TM^), of less than 10 nm radius of curvature (typically 8 nm), ~125 μm length, ~30 μm width, ~42 N/m force constant and ~330 kHz resonance frequency. The AFM images were processed with XEI program (v 1.8.0—Park Systems) for displaying purpose and roughness evaluation. In order to improve the topographic details, the images are presented in so-called “enhanced contrast” view mode. This is a peculiarly colored view mode patented by Park Systems used to enhance the morphological characteristics, which employ the change of a pixel relative to its neighbors. Representative line scans are showed below 2D AFM images, which present in detail the surface profile of the scanned samples. The root-mean-squared roughness (Rq) represents the standard deviation of the height value in the image, while the peak-to-valley parameter (Rpv) is the height difference between the lowest and the highest points [27].

#### 2.3.4. Diffuse Reflectance UV-Vis

UV–visible spectra were obtained with a Perkin Elmer Lambda 35 spectrophotometer equipped with an integrating sphere. Using the Kubelka–Munk function, the recorded reflectance data were changed into absorption spectra.

#### 2.3.5. Reactive Oxygen Species (ROS) Generation under Visible Light Irradiation

*Hydroxyl radicals (*•OH*) assessment*

In order to evaluate the hydroxyl radical’s production (•OH), a coumarin solution (10 mM, Merck) containing 0.001 g of suspended powder (inorganic and hybrid complex) was irradiated with visible solar light (Peccel Solar Simulator, Japan, equipped with a cut-off filter λ > 420 nm, Asahi Spectra). The spectrum of the illumination source is provided in Supplementary Information (Figure S1). The fluorescent umbelliferone resulted from coumarin interaction with the photogenerated hydroxyl radicals and was further monitored with a Carry Eclipse fluorescence spectrometer, Agilent Technologies, (slits set to 5/10 nm in excitation and emission) for λ_exc_ = 330 nm.

*Superoxide anion O_2_^•−^ assessment*

For O_2_^•−^ trapping, 0.003 g powder (inorganic and hybrid complex) was dispersed in 3 mM solution of 2,3-Bis(2-methoxy-4-nitro-5-sulfophenyl)-2*H*-tetrazolium-5-carboxanilide (XTT sodium salt) and exposed to simulated solar light. The interaction of XTT sodium salt with photogenerated O_2_^•−^ leads to XTT formazan formation evidenced by a broad peak with a maximum located at 470 nm. An Analytik Jena Specord 200 Plus spectrophotometer was used for the UV–visible spectra recording.

#### 2.3.6. X-ray Fluorescence (XRF) Characterization for Ion Releasing Measurements

XRF was used for elemental analysis of the liquid collected after a dialysis process involving the suspended powders in water (ZnSe, ZnSe-TiO_2_, TiO_2_, Lys/ZnSe, Lys/ZnSe-TiO_2_ and Lys/TiO_2_). In this sense, 0.03 g of each material was dispersed in 2 mL ultrapure water (Millie-Q system, >18 MΩcm), put in a dialysis bag (ZelluTrans/Roth regenerated cellulose membrane with wall thickness of 28 μm) and immersed in 5 mL ultrapure water, (Millie-Q system) contained by a glass beaker. The suspension was gently shaken with a magnetic stirrer for 24 h at room conditions. The system was adapted after Balint et al. [28]. Aliquots from the dialyzed liquid were subjected to XRF analysis looking for the released ions (presumable zinc, selenium and titanium ions). The measurements were performed using a Rigaku ZSX Primus II spectrometer (Rigaku Corp., Tokyo, Japan), equipped with 4.0 kW X-ray Rh tube. EZ-scan combined with Rigaku SQX fundamental parameters software (standard less) was used for data analysis.

For the liquid samples, the droplet method was used. Droplet method (or filter paper method) is used for analysis of the dried droplet solution on special filter paper [29].

### 2.4. Antimicrobial Activity Assay

#### 2.4.1. Antimicrobial Activity Assay for the Inorganic Samples (ZnSe, ZnSe-TiO_2_ and TiO_2_) and Their Hybrid Complex with Lysozyme (Lys/ZnSe, Lys/ZnSe-TiO_2_ and Lys/TiO_2_)

*Staphylococcus aureus* ATCC 25923 (American Type Culture Collection) and *Micrococcus lysodeikticus* ATCC 4698 lyophilized cells (from Lysozyme Activity Kit—LY0100 Sigma Aldrich) were used as model organisms to evaluate the antibacterial activity of target materials. The reference strains were grown overnight on TSB (Tryptic Soy Broth, Scharlab, Spain) at 37 °C. The antibacterial potential was assessed based on the pour plate method. The fresh bacterial inoculum was suspended in 0.85% NaCl and the turbidity was adjusted to 10^8^ colony-forming units per milliliter (CFU/mL), corresponding to 0.5 Mc Farland standard, according to CLSI (Clinical and Laboratory Standards Institute). The standardized bacterial suspension was inoculated into TSB and incubated for four hours at 37 °C with 150 rpm shaking in the presence of 0.01 g of the synthesized samples. Subsequently, 10-fold serial dilutions were performed from the obtained suspension and appropriate volumes of diluted samples were spread on TSA (Tryptic Soy Agar, Scharlab, Spain) plates and incubated at 37 °C for 24 h. The number of colonies were counted and the results were expressed as CFU/mL (colony-forming unit per mL). The percentage of cell survival was calculated based on the control sample represented by the untreated bacterial culture. Experiments were performed in triplicate.

#### 2.4.2. Antimicrobial Activity Assay for the Samples of Interest Assisted by Visible Light Irradiation (λ > 420 nm)

The antibacterial activity of the synthesized materials was tested against the reference bacterial strains *Staphylococcus aureus* ATCC 25923 and *Micrococcus lysodeikticus* ATCC 4698. Cells were grown overnight at 37 °C with 150 rpm shaking by inoculating a bacterial single colony in TSB. A bacterial suspension obtained in MilliQ sterile water with a concentration of 10^5^ colony-forming units per milliliter (CFU/mL) was used for the antibacterial experiment. The efficacy of the samples against bacterial suspension was assessed in a sterile 96-well plate. A total of 0.01 g from the investigated materials (ZnSe, ZnSe-TiO_2_, TiO_2_, Lys/ZnSe, Lys/ZnSe-TiO_2_ and Lys/TiO_2_) was placed in each well, over which 20 µL of standardized bacterial suspension was added and irradiated with visible light (λ > 420 nm) for 10, 20 and 30 min. Subsequently, fresh liquid culture medium was added to each sample well. A serial decimal dilution in a sterile saline solution was performed and plated onto TSA. The number of colonies was counted after 24 h of incubation at 37 °C and expressed as CFU/mL. A control sample, represented by the irradiated bacterial suspension without materials of interest, was also prepared. The percentage of cell survival was calculated. Experiments were performed in triplicate. After 30 min of light exposure, 3 µL of each bacterial suspension containing ZnSe, ZnSe-TiO_2_, TiO_2_, Lys/ZnSe, Lys/ZnSe-TiO_2_ and Lys/TiO_2_ was extracted and deposited on glass substrate (Heinz Herenz, Hamburg, Germany) for AFM analysis.

## 3. Results and Discussion

### 3.1. Scanning Electron Microscopy (SEM)

**Figure 1:**
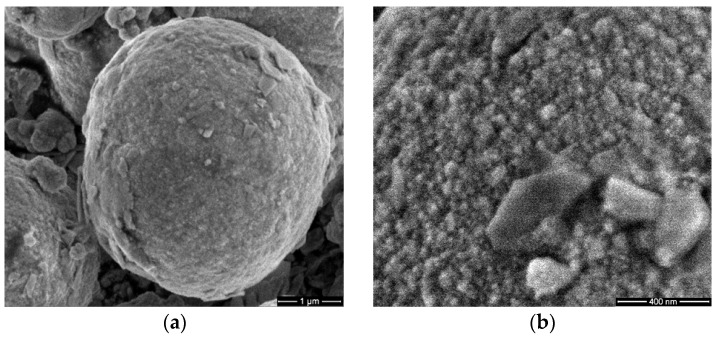

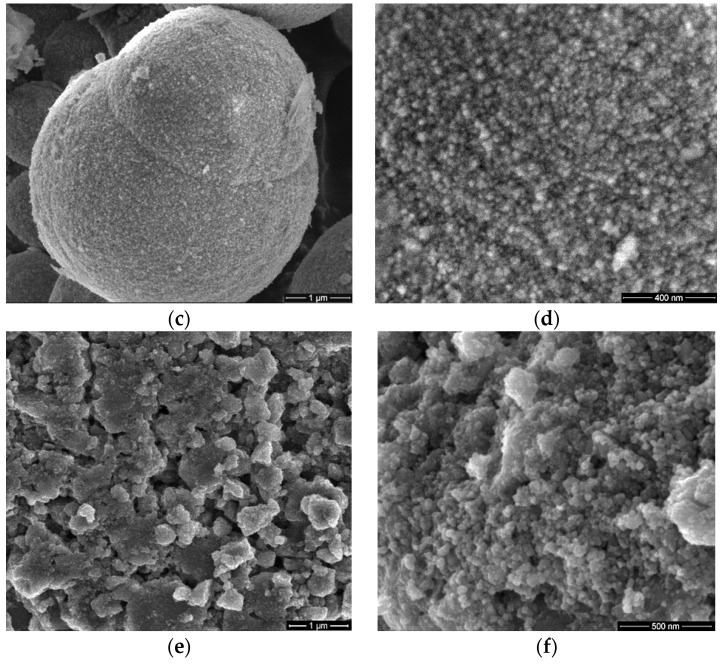
SEM images of ZnSe (**a**,**b**), ZnSe-TiO_2_ (**c**,**d**) and TiO_2_ (**e**,**f**).

SEM images of the ZnSe, ZnSe–TiO_2_ and TiO_2_ samples were presented in Figure 1, which shows that nanoparticles assembled as micrometer-sized spheres for ZnSe-based materials and aggregates of nano-scaled particles for TiO_2_. A perceivable surface roughness and incipient exfoliation process can be observed for ZnSe spheres (Figure 1a,b), whereas for the composite material (ZnSe–TiO_2_), smaller and closely packed particles can be depicted at the surface of microspheres with disposition for gathering (Figure 1c,d). 

These morphological changes are induced by the presence of TiO_2_ at the surface of the composite material (ZnSe-TiO_2_), which been shown in our previous work [17]. This can trigger a specific antibacterial activity of the composite sample (ZnSe-TiO_2_) relative to the bare ones (ZnSe, TiO_2_). Figure 1e,f shows aggregates formed by TiO_2_ nanoparticles ranged between 50 and 100 nm.

### 3.2. Dark Field Microscopy (Cyto Viva)

**Figure 2:**
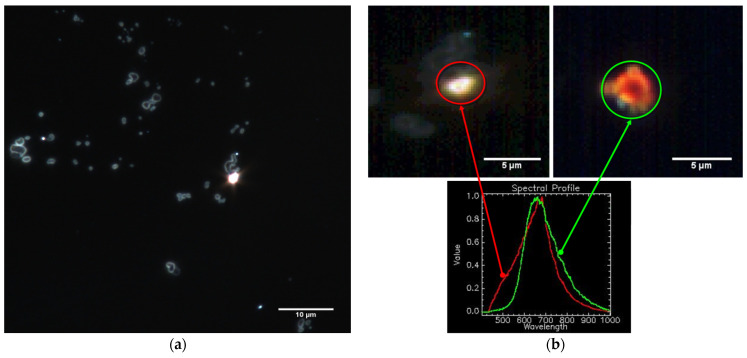
Global optical image (100×) (**a**) and hyperspectral images (100× + 4× digital zoom; lysozyme /ZnSe-TiO_2_ (green circle) *Staphylococcus aureus*/Lys/ZnSe-TiO_2_ (red circle) and their spectral analysis—bottom)) (**b**) of *Staphylococcus aureus* contacting the Lys/ZnSe-TiO_2_ complex.

Dark field microscopy was used for illustrating the interaction of *Staphylococcus aureus* with the lysozyme/ZnSe-TiO_2_ complex (Figure 2).

Figure 2a presents the bacterial morphology after its exposure to the Lys/ZnSe-TiO_2_ complex (according to the Section 2.3.2). Additionally, in this image, the hybrid complex appears as a sparkling point due to the fluorescence of the lysozyme (containing the tryptophan residues) that surrounds the inorganic particle (ZnSe-TiO_2_) and generates a core shell-type structure (this can be clearer depicted in Figure 2b, green circle) and is also proven in previous work [17]. Bacteria fragments can be observed in Figure 2a proving the harmful effect of Lys/ZnSe-TiO_2_ on *Staphylococcus aureus*. The spectral comparison from Figure 2b certifies the contact of bacteria with the Lys/ZnSe-TiO_2_ complex by providing the mean spectral profile for both lysozyme/ZnSe-TiO_2_ (green signaled) and the *Staphylococcus aureus*/lysozyme/ZnSe-TiO_2_ (red signaled) in the 400–1000 nm spectral range.

A mean region of interest (ROI) was captured from both samples. The data were then normalized to unity for comparison, being obvious that the lysozyme/ZnSe-TiO_2_ hybrid complex and *Staphylococcus aureus*/lysozyme/ZnSe-TiO_2_ exhibit a unique and distance spectral profile in the visible–NIR range (400–1000 nm), with the *Staphylococcus aureus*/lysozyme/ZnSe-TiO_2_ having a peak location approximately 25 nm shifted to the right. Briefly, Figure 2a,b emphasizes the antibacterial effect of the lysozyme/ZnSe-TiO_2_ complex against *Staphylococcus aureus*. In this sense, the effect may be attributed to the bacteria contact with lysozyme/ZnSe-TiO_2_ (certified by Figure 2b), but other antibacterial mechanisms (ROS generation and ion releasing) can be envisaged. The foregoing antibacterial assay may add new data regarding the synergetic effects of lysozymes and ZnSe-TiO_2_. 

### 3.3. Atomic force microscopy

**Figure 3:**
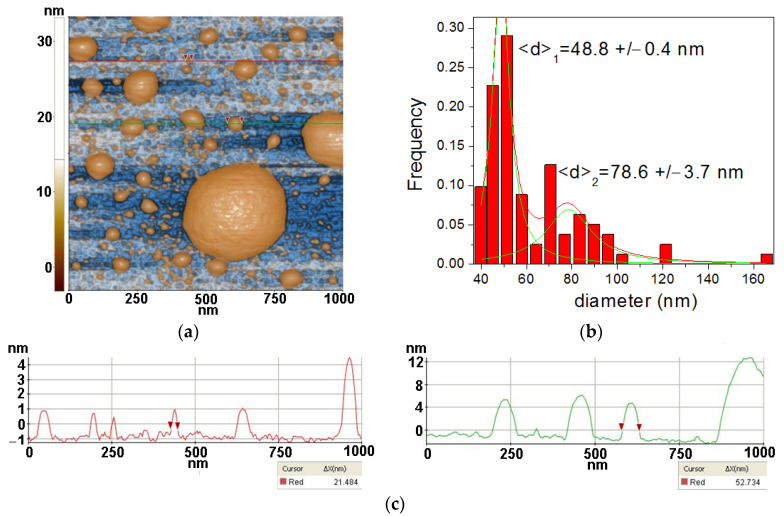
Two-dimensional AFM image scanned at a scale of (1 µm × 1 µm) of TiO_2_ deposited on bare Si substrate (**a**) together with a particles’ diameters histogram for the TiO_2_, fitted with two Gaussian distribution functions (**b**) and two characteristic surface profiles (red and green line scans) (**c**).

**Figure 4:**
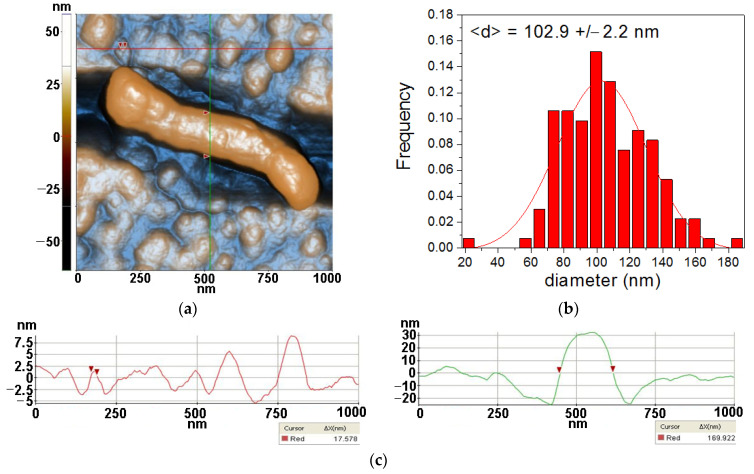
Two-dimensional AFM image scanned at the scale of (1 µm × 1 µm) for lysozyme deposited on bare Si substrate (**a**), particles’ diameters histogram for lysozyme deposited on Si fitted with a Gaussian distribution function (**b**) and two characteristic surface-profiles (line scans), horizontal and vertical (**c**).

**Figure 5:**
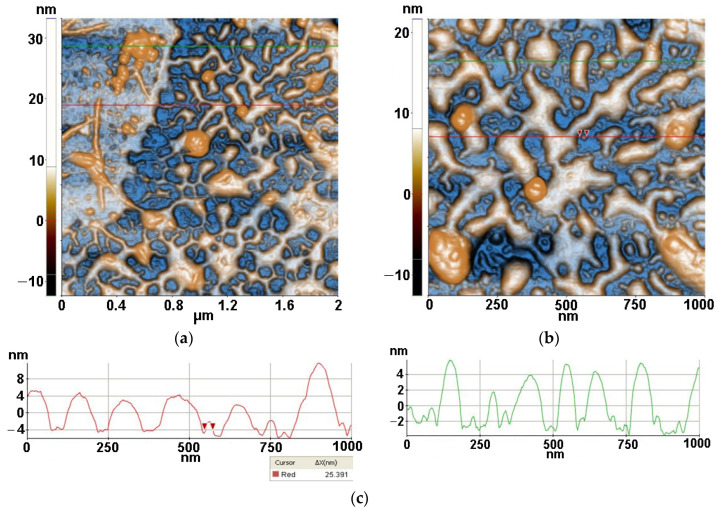
Two-dimensional AFM image for lysozyme deposited on TiO_2_/Si substrate, scanned at the scale of (2 µm × 2 µm) (**a**) and at the scale of (1 µm × 1 µm) (**b**), together with two horizontal characteristic surface profiles (line-scans) (**c**), plotted from (**b**).

**Figure 6:**
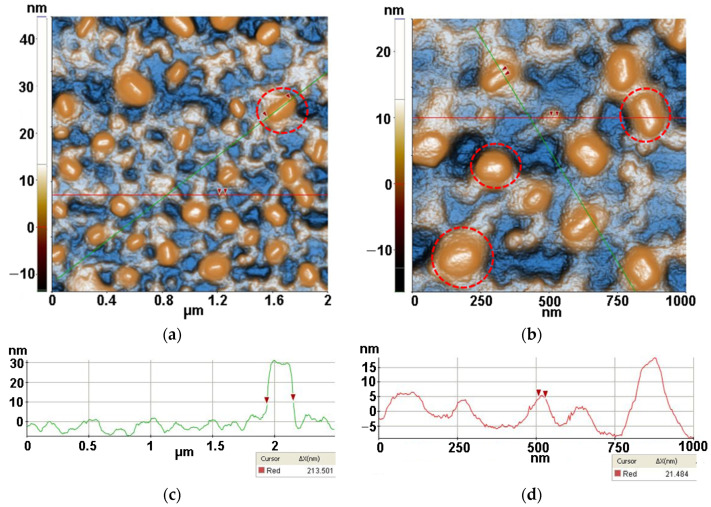
Two-dimensional AFM images for MC (from buffered suspension) deposited on Lys/TiO_2_/Si substrate, recorded at the scale of (2 µm × 2 µm) (**a**) and at the scale of (1 µm × 1 µm) (**b**), together with horizontal characteristic surface profiles (line-scans) collected from (**a**) green line (**c**), and from (**b**) red line (**d**).

Firstly, the AFM images were captured for TiO_2_ nano-scaled particles supported on Si substrates alone and together with lysozyme (Lys) and *Micrococcus lysodeikticus* (MC), collected from buffered suspensions. Figure 3, Figure 4, Figure 5 and Figure 6 contain AFM images recorded 48 h after the sample preparation.

The TiO_2_ powder was imaged by dispersing it in water by ultrasonication (at a concentration of 0.5 mg/mL) and deposited on a clean Si(100) substrate. Figure 3 presents the morphology of the TiO_2_ at the scale of (1 µm × 1 µm). From Figure 3, it can be observed that the TiO_2_ powder prepared by sol–gel method plus further hydrothermal tratment exhibits a majority population of small hemi-spherical particles, with a main diameter around 50 nm and a tendency to form agglomerated particles with diameters up to hundreds of nm in diameter. The tendency for nanoparticles to agglomerate in spherical aggregates can also be noticed. At the scale of (1 µm × 1 µm), as exemplified in Figure 3, the TiO_2_ particles deposited on Si(100) are characterized by the following roughness parameters: Rq = 6.51 nm and Rpv = 36.44 nm.

From Figure 4, two morphological characteristics can be observed for lysozymes deposited on Si, namely the quasi-spherical and fibrillar shaped species. More than that, it can be noticed that the features with a quasi-spherical (doughnut) appearance are composed of smaller entities, such as that marked between the two red arrows along the horizontal red line, with a diameter of ~17.6 nm. On the other hand, the diameter of the fibrillar structure is ~170 nm, as suggested by the vertical green line, while its length is ~800 nm. Nevertheless, its texture suggests that the fiber is formed by the agglomeration of individual lysozymes. At the scale of (1 µm × 1 µm) (Figure 4) the Lys/Si system is characterized by the following roughness parameters: Rq = 17.11 nm and Rpv = 122.34 nm.

Figure 5 presents the AFM images obtained for the Lys/TiO_2_/Si system (resulted after the drop-casting deposition of lysozymes onto TiO_2_/Si specimens). From the 2D AFM images in Figure 5, it can be seen that lysozymes cover the TiO_2_ particles exhibiting a strong tendency to form a network of inter-connected fibrils which are ~6–8 nm in height. Random individual TiO_2_ particles are still visible (some of them gathered in form of clusters—as seen in the corner left image of Figure 5b between the lysozyme fibers as well evidenced by the AFM image recorded at the scale of (2 µm × 2 µm) (Figure 5a). At the scale of (1 µm × 1 µm), as seen in Figure 5b, the Lys/TiO_2_ particles deposited on Si(100) are characterized by the following roughness parameters: Rq = 4.24 nm and Rpv = 34.20 nm. The deposition of lysozymes takes place mainly on the TiO_2_ particles, coating them and favoring fibrillar depositions.

*Micrococcus lysodeikticus* (MC) was added from buffered cell suspension to the lysozyme previously deposited on the TiO_2_/Si substrate resulting in the morphology presented in Figure 6 (MC/Lys/TiO_2_/Si). The previously formed network-like Lys/TiO_2_ system that the MC bacteria it is disposed on top of, in the form of compact hills, can be seen marked by red circles in Figure 6. The MC pattern is even more recognizable at the scale of (2 µm × 2 µm), where diameters of ~200 nm are suggested by the corresponding line-scan from Figure 6c. At larger scales (4 µm × 4 µm and 8 µm × 8 µm—not shown here), MC bacteria with larger diameters (3–400 nm) are present on Lys/TiO_2_/Si. Taking into account that MC is deposited from buffered solution (not from bacterial inoculum) and the images were recorded after 48 h from deposition, these AFM images can be considered relevant for the MC interaction with lysozymes. The proper dimension of the MC will be revealed starting with Figure 8.

From the corrugation point of view, at the scale of (1 µm × 1 µm) (see Figure 6b), the MC/Lys/TiO_2_ deposited on Si(100) is characterized by the following roughness parameters: Rq = 6.50 nm and Rpv = 41.30 nm.

**Figure 7:**
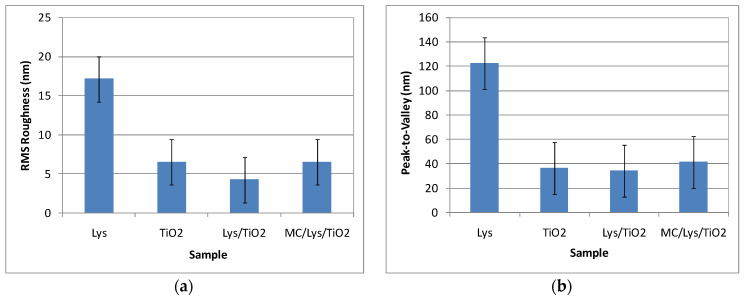
Corrugation characteristics of all scanned systems: RMS roughness (**a**) and peak-to-valley (**b**). Evaluated from AFM images measured after 48 h.

The roughness behavior of all scanned systems is summarized in Figure 7. It can be observed that the most corrugated surface is seen in the case of Lys deposited on Si due to the fibrillar-shaped particles and their agglomeration in the form of clusters.

**Figure 8:**
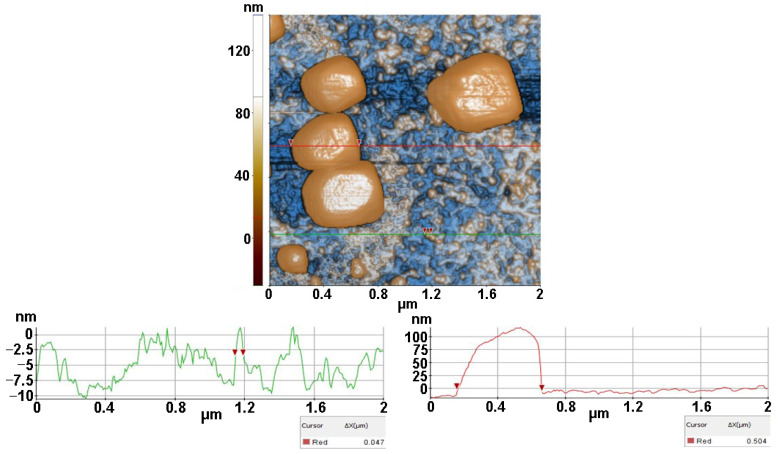
Two-dimensional AFM image of MC (*M. lysodeikticus* from bacterial inoculum) with TiO_2_ deposited on glass substrate, recorded at the scale of (2 µm × 2 µm) with two characteristic surface profiles (red and green line scans).

The next set of AFM images were obtained by depositing a drop of suspension containing the bacterial inoculum (prepared according to Section 2.4.2) and inorganic matrices (TiO_2_, ZnSe-TiO_2_) on clean microscopic glass substrates and measured after 1 h from specimen preparation. Figure 8 presents the MC bacteria from inoculum and TiO_2_ deposited on glass. 

The massive blocks represent the random disposed MC bacteria as having a particular texture. The red line suggests a diameter of ~500 nm, while the small particles are TiO_2_ with diameters one order of magnitude smaller (~50 nm, as suggested by the particle selected along the green line). At the scale of (2 µm × 2 µm) (Figure 8) the MC/TiO_2_/glass sample is characterized by the following roughness parameters: Rq = 39.47 nm and Rpv = 172.75 nm.

**Figure 9:**
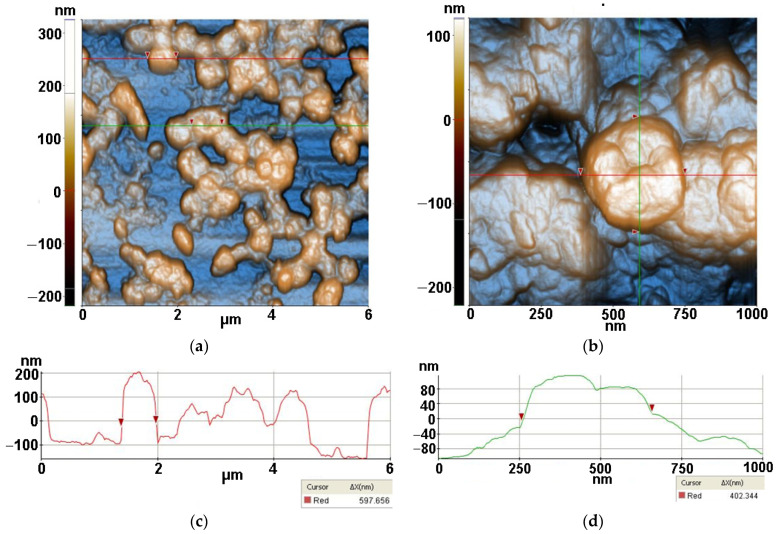
Two-dimensional AFM images of MC (from inoculum) with Lys/TiO_2_ deposited on glass substrate, recorded at the scale of (6 µm × 6 µm) (**a**) and (1 µm × 1 µm) (**b**), with one characteristic surface profile for each scale: 6 µm red (**c**) and 1 µm green (**d**).

Figure 9 presents the MC bacteria with Lys/TiO_2_ deposited on glass at the scales of (6 µm × 6 µm) (Figure 9a) and (1 µm × 1 µm) (Figure 9b). The massive shape of the MC bacteria prevails over the morphology of Lys/TiO_2_, with the features selected along the red line being ~600 nm and along the vertical green line of approx. 400 nm. Nevertheless, the texture of MC is visible, and the dimensions are influenced by the presence of Lys/TiO_2_. At the scale of (2 µm × 2 µm), not shown here, the MC/Lys/TiO_2_/glass sample is characterized by the following roughness parameters: Rq = 73.36 nm and Rpv = 512.61 nm.

**Figure 10:**
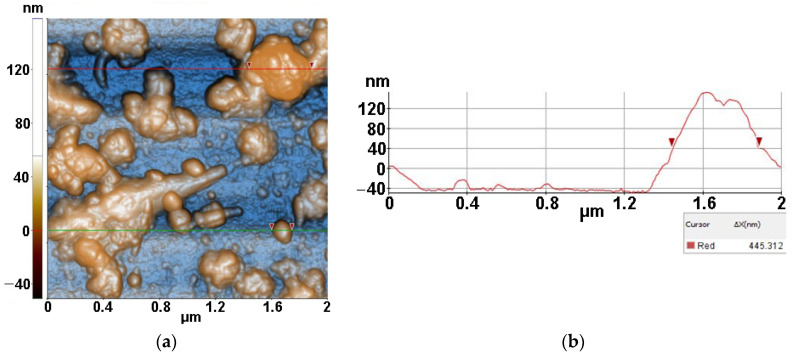
Two-dimensional AFM image of MC from inoculum with ZnSe after 30 min of irradiation deposited on glass substrate, recorded at the scale of 2 µm × 2 µm (**a**), together with a characteristic line scan (**b**).

Figure 10 presents AFM image of MC from inoculum with ZnSe after 30 min of irradiation, deposited on glass substrate, recorded at the scale of 2 µm × 2 µm, showing one distorted MC bacteria (upper right corner) with a diameter of approx. 445 nm. This sample is characterized by the following roughness parameters: Rq = 28.22 nm and Rpv = 208.80 nm.

**Figure 11:**
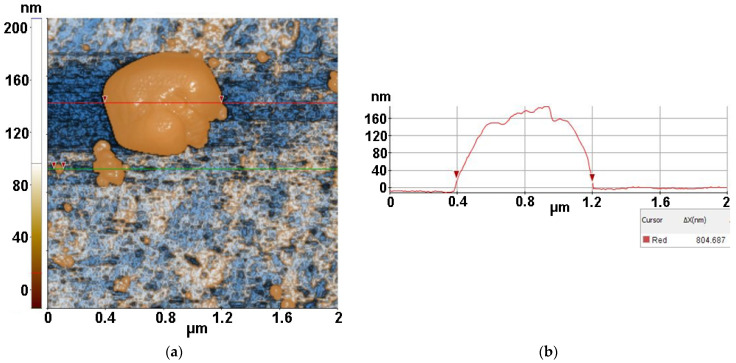
Two-dimensional AFM image of MC from inoculum with ZnSe-TiO_2_ after 30 min of irradiation, deposited on glass substrate, recorded at the scale of 2 µm × 2 µm (**a**), together with a characteristic line scan (**b**).

Finally, Figure 11 presents an AFM image of MC from inoculum with ZnSe-TiO_2_ after 30 min of irradiation deposited on glass substrate, recorded at the scale of 2 µm × 2 µm. The image shows one massive MC bacterium with a diameter of ~800 nm, which is surrounded by some random fragments resulting from the MC interaction with ZnSe-TiO_2_. The MC/ZnSe-TiO_2_ sample is characterized by the following roughness parameters: Rq = 42.76 nm and Rpv = 221.41 nm.

**Figure 12:**
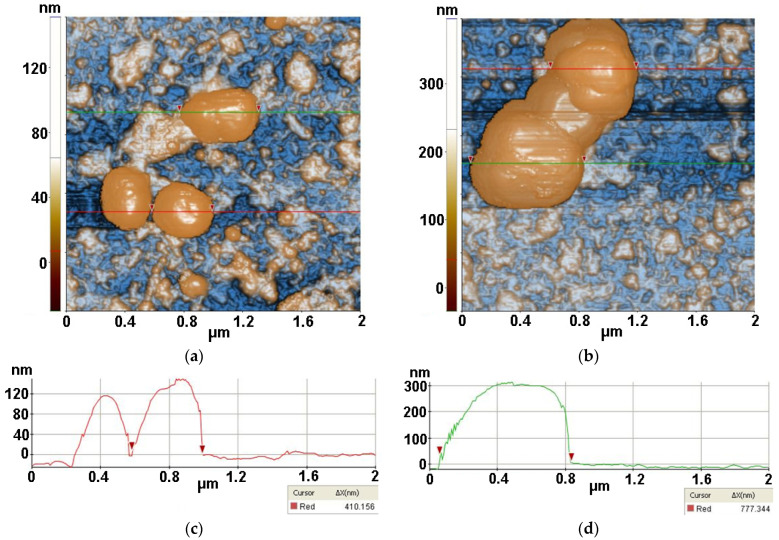
Two-dimensional AFM images of *S. aureus* (from inoculum) with TiO_2_ deposited on glass substrate, after 30 min of irradiation, recorded at the scale of (2 µm × 2 µm) in two different areas (**a**,**b**), with two characteristic surface profiles for each image in red (**c**) and green (**d**).

Figure 12 presents the interaction of *S. aureus* bacteria (from inoculum) with TiO_2_ material after 30 min of irradiation. Figure 12a shows three *S. aureus* bacteria, the selected one having a diameter of ~410 nm, while Figure 12b catches an agglomeration of bacteria, the largest part being of ~777 nm (along the green line). 

The *S. aureus*/TiO_2_ sample from Figure 12a is characterized by the following roughness parameters: Rq = 29.34 nm and Rpv = 181.22 nm.

**Figure 13:**
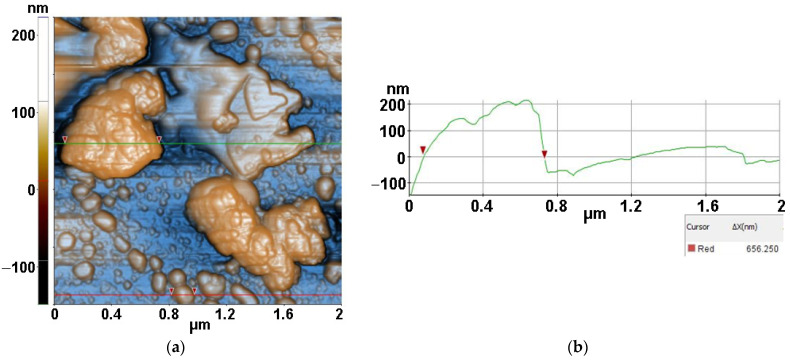
Two-dimensional AFM images of *S. aureus* (from inoculum) with ZnSe deposited on glass substrate, after 30 min of irradiation, recorded at the scale of (2 µm × 2 µm) (**a**), with a characteristic surface profile (**b**).

Figure 13 presents the interaction of *S. aureus* bacteria with ZnSe material after 30 min of irradiation. The image captured a bacterial agglomeration of approx. 656 nm (selected between two red arrows along the green line) nearby a ZnSe fragment. In the lower half of the image, some other bacterial fragments, separated or gathered, are visible. The *S. aureus*/ZnSe sample from Figure 13 is characterized by the following roughness parameters: Rq = 52.61 nm and Rpv = 371.41 nm.

**Figure 14:**
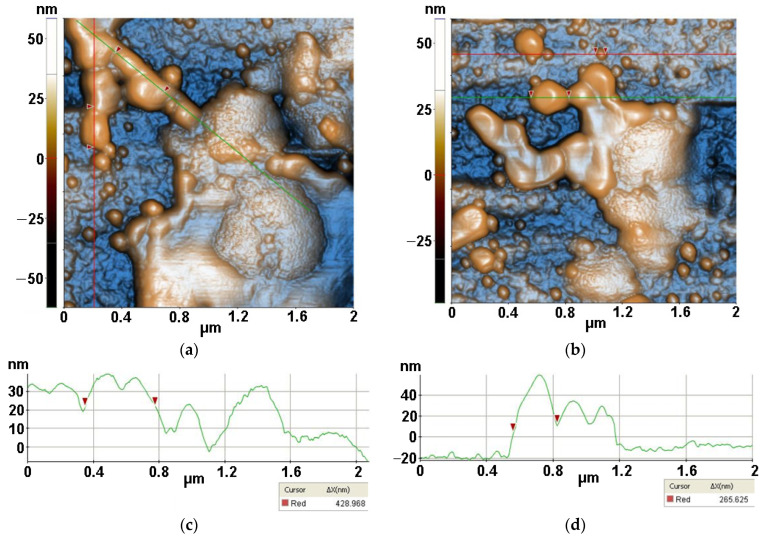
Two-dimensional AFM images of *S. aureus* (from inoculum) with ZnSe-TiO_2_ deposited on glass substrate, after 30 min of irradiation, recorded in two areas at the scale of (2 µm × 2 µm) (**a**,**b**), with two characteristic surface profiles (**c**,**d**).

Finally, Figure 14 presents AFM images recorded for *S. aureus* (from inoculum) interaction with ZnSe-TiO_2_ deposited on glass, after 30 min of irradiation, at the scale of (2 µm × 2 µm). The two AFM images captured distorted *S. aureus* bacteria with diameters from 428 nm (a) to 265 nm (b) after interaction with ZnSe-TiO_2_ material. The ZnSe-TiO_2_ is visible as fragments with a peculiar texture. Figure 14a is characterized by the following roughness parameters: Rq = 17.98 nm and Rpv = 120.79 nm.

**Figure 15:**
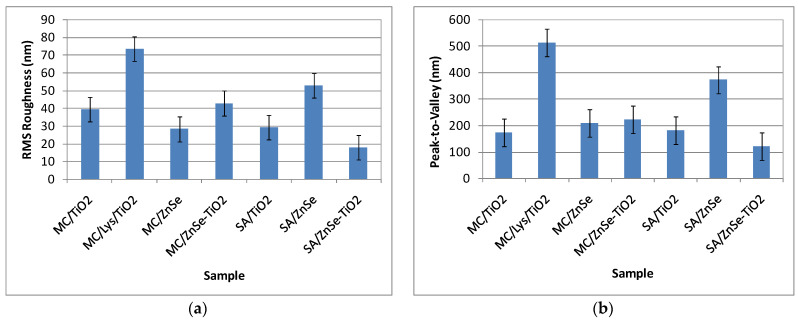
Corrugation characteristics of all scanned systems: RMS roughness (**a**) and peak-to-valley (**b**) from inoculum, evaluated from AFM images measured after 1 h.

For all samples deposited on glass, the roughness behavior of all scanned AFM images is summarized in Figure 15 showing that the most corrugated surface is observed for the MC/Lys/TiO_2_ system. 

### 3.4. UV-Vis Spectroscopy 

**Figure 16:**
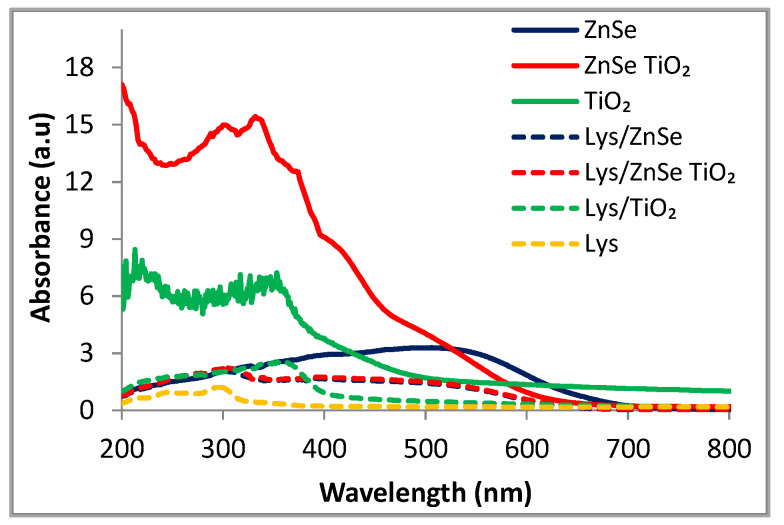
Comparative UV–visible spectra of ZnSe, ZnSe-TiO_2_ and TiO_2_ powders before and after lysozyme loading.

The UV–visible analysis was performed on inorganic ZnSe, ZnSe-TiO_2_, TiO_2_ and lysozyme modified materials. The characterization of the inorganic samples was meant to depict the light absorptive properties of the samples revealing their structural particularities. The spectra of interest were collected in the range of 200–800 nm (Figure 16) and showed the higher light absorption intensity for the ZnSe-TiO_2_ composite relative to the pristine samples, both in UV and visible domains. ZnSe material has the broad absorption band ranging between 400 and 600 nm.

Although the present TiO_2_ sample is in line with the TiO_2_ reference materials, having the absorption edge around 400 nm, it also displays a long and well-defined absorption tail in the visible range attributable to the generated surface defects during the post-synthesis thermal treatment.

Figure 16 reveals the UV–visible spectra for the hybrid complexes with lysozyme of the inorganic samples and the bare lysozyme. Although the decreasing absorbance is obvious for all the modified samples, Lys/ZnSe and Lys/ZnSe-TiO_2_ still preserve light absorptive capacity in the visible range (400–600 nm).

### 3.5. ROS Generation under Visible Light Irradiation (λ > 420 nm)

**Figure 17:**
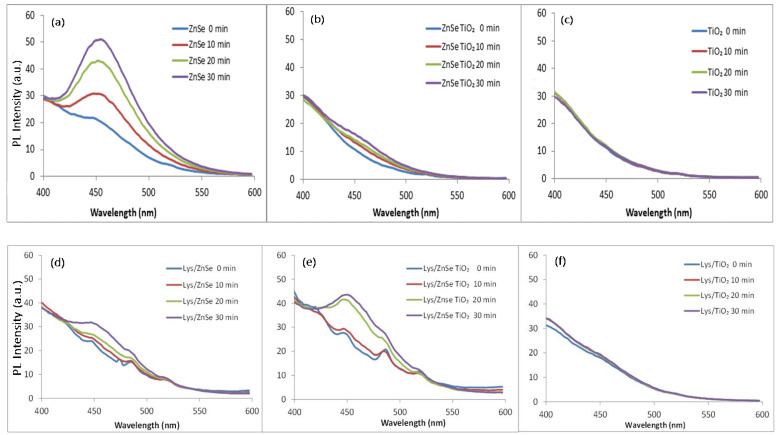
Generation of hydroxyl radical by inorganic (**a**–**c**) and lysozyme modified materials (**d**–**f**) under visible light irradiation.

Hydroxyl radical (•OH) generation was evaluated from PL emission at 451 nm for λ_exc_ = 330 nm, this being attributed to the formation of umbelliferone by the interaction of coumarin with the photogenerated •OH [30]. Figure 17a shows the ability of the ZnSe material to generate hydroxyl radicals under visible light irradiation that almost disappears when TiO_2_ is added to composite materials (Figure 17b).

The TiO_2_ sample proves to be totally inactive to generate •OH radicals under visible light (Figure 17c), while the ZnSe-TiO_2_ sample shows a very slight activity too. Figure 17 shows that the antibacterial mechanistic pathways of ZnSe sample may involve •OH. Figure 17d–f also present the formation of •OH radicals under visible light irradiation by the lysozyme modified samples, which appears to be sharply decreased for the ZnSe sample. On the contrary, the capacity of Lys/ZnSe-TiO_2_ to generate •OH relative to the unmodified sample seems to be higher with a maximum centered at 451 nm appearing after 20 and 30 min of light exposure. In the case of the Lys/TiO_2_ sample, this is missing.

The superoxide anion (O_2_^•−^) formation was monitored following the intensity of the formazan characteristic peak (485 nm) [31].

**Figure 18:**
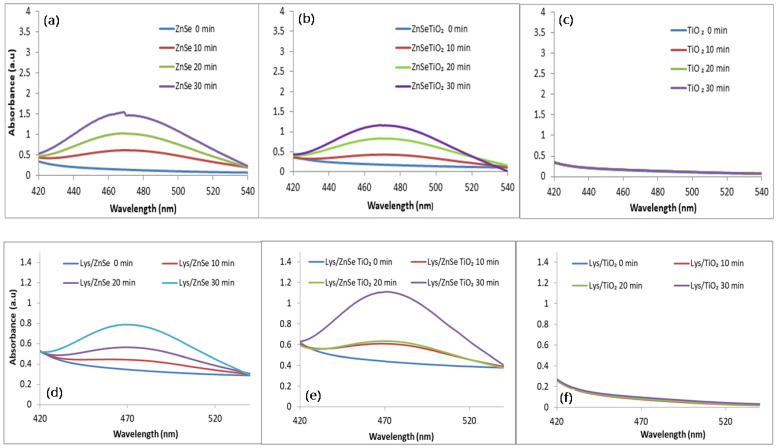
The time course of O_2_^•−^ formation over inorganic (**a**–**c**) and lysozyme modified samples (**d**–**f**) under visible light irradiation.

Figure 18a,b displays broad absorption bands for ZnSe and ZnSe-TiO_2_ samples that indicate presumable antibacterial effects due to the presence of O_2_^•−^. Conversely, the TiO_2_ material shows a lack of activity. This could be related to the presence of surface defects induced by the hydrothermal treatment and the known TiO_2_ inactivity in the visible range.

For the lysozyme modified samples, a well-defined characteristic band with a maximum at 470 nm is displayed in the case of Lys/ZnSe-TiO_2_ and Lys/ZnSe. This certifies the production of O_2_^•−^ despite the presence of adsorbed lysozyme on the inorganic surfaces. This seems to allow the electron trapping by the adsorbed oxygen.

### 3.6. Antimicrobial Activity Assay for the Inorganic Samples (ZnSe, ZnSe-TiO_2_, TiO_2_) and Their Hybrid Complex with Lysozyme (Lys/ZnSe, Lys/ZnSe-TiO_2_ and Lys/TiO_2_) against S. aureus

**Figure 19:**
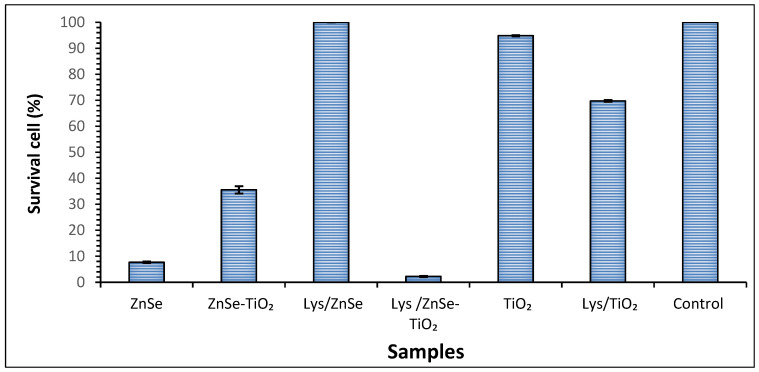
Survival cell percentage of *S. aureus* ATCC 25923 after four hours of exposure to synthesized materials. The data are presented as the mean of three independent measurements, with error bars representing the standard deviation of the mean.

In order to determine the potential antimicrobial effect of the synthesized materials, we assessed the planktonic growth of microbial cells in the presence of investigated samples by using the viable cell count method. Our study showed that the planktonic growth of the *S. aureus* bacterial strain was influenced differently by the investigated samples as follows: it was significantly inhibited in the presence of ZnSe-based materials after four hours of exposure, as compared to the control sample but slightly affected by the presence of TiO_2_. Namely, a significant bacterium growth inhibition was observed when the cells were incubated in the presence of a ZnSe, ZnSe-TiO_2_ and Lys/ZnSe-TiO_2_ samples as reflected by the survival cell percentages (7.6%, 35.5%, and 2.2%, respectively) (Figure 19). Surprisingly, the sample Lys/ZnSe proves to be inactive against *S. aureus*, but on the contrary, a beneficial effect of adding lysozymes to the ZnSe-TiO_2_ sample is achieved.

In order to explain the strange behavior of the Lys/ZnSe sample, one of the most cited antibacterial mechanisms, namely the ion releasing, was investigated (according to Section 2.4) by X ray fluorescence measurements. Zinc ion release in solution through a dialysis membrane was proven only for the ZnSe sample. In the same experimental conditions, this is missing for the Lys/ZnSe and the other samples (Supplementary Materials Figure S2). This result could explain the lack of activity for the Lys/ZnSe sample if the ion release is considered the main degradative mechanism against S. aureus and for the high antibacterial activity of the bare ZnSe sample. It is likely that the lysozyme’s presence at the ZnSe surface hinders the release of zinc ions in solution. Based on these results, different mechanisms (i.e., ROS generation) should be considered decisive for the antibacterial activity of the other samples.

The inhibition of ZnSe reactivity towards S. aureus induced by lysozyme loading was checked taking into account the different amounts of lysozyme: 0.01 g powder and 20 μL of initial lysozyme suspension added to ZnSe led to 90% cell survival; and 100% cell survival being obtained by increasing the amount of available lysozyme by ten and fifty times. This particular behavior of Lys/ZnSe towards *S. aureus* needs to be closely investigated in a future dedicated study.

### 3.7. Antimicrobial Activity Assay for the Inorganic Samples (ZnSe, ZnSe-TiO_2_ and TiO_2_) and Their Hybrid Complex with Lysozyme (Lys/ZnSe, Lys/ZnSe-TiO_2_ and Lys/TiO_2_) against M. lysodeikticus

**Figure 20:**
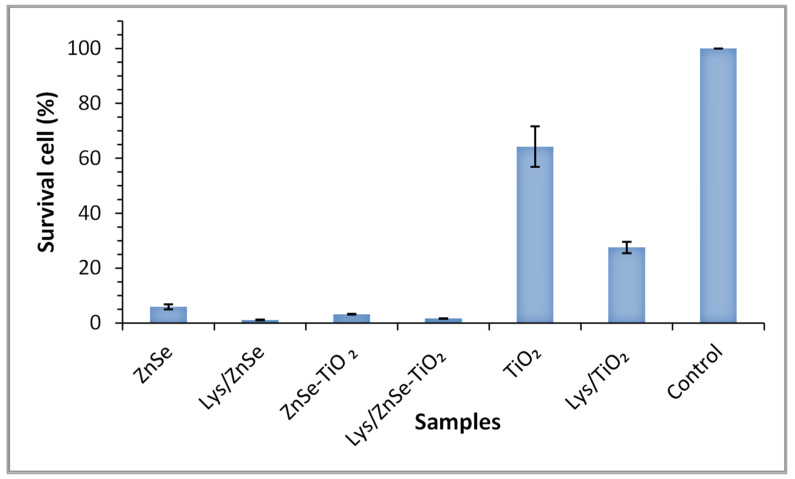
Cell survival percentage of *Micrococcus lysodeikticus* ATCC 4698 after four hours of exposure to synthesized materials. The data are presented as the mean of three independent measurements with error bars representing the standard deviation of the mean.

The antibacterial effects of the various synthesized materials were investigated by comparing the number of viable Micrococcus cells after being in contact with the target samples for four hours (Figure 20).

Analyzing the data depicted in Figure 20, it can be observed that the number of viable *Micrococcus* cells considerably decreased in the presence of ZnSe and ZnSe-TiO_2_ samples when compared with the untreated sample, resulting in a bacterial reduction of 94.13% for ZnSe and 98.91% for ZnSe-TiO_2_. Viability was also impaired after four hours of incubation of the microbial cells in the presence of TiO_2_ (35.8% bacterial reduction) relative to the control cells. 

Further analysis of the lysozyme loaded materials revealed their important role in the inhibition of microbial growth, displaying higher antibacterial activity than lysozyme-free samples. Figure 20 shows the decreasing (by 50%) viability M. lysodeikticus cells with lysozyme addition to the TiO_2_ relative to the bare sample. 

### 3.8. Antibacterial Activity of the Inorganic and Hybrid Materials (ZnSe, ZnSe-TiO_2_, TiO_2_ Lys/ZnSe, Lys/ZnSe-TiO_2_ and Lys/TiO_2_) Exposed to Visible Light Irradiation against S. aureus and M. lysodeikticus 

**Figure 21:**
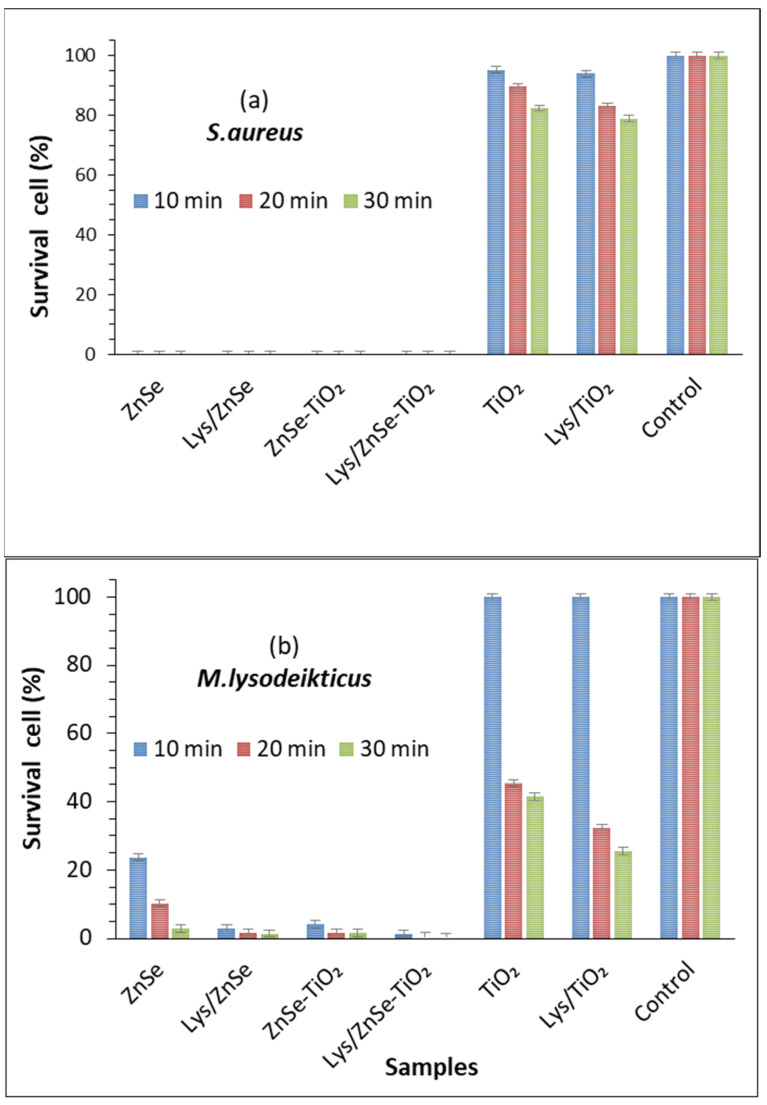
Cell survival percentage of *S. aureus* ATCC 25923 (**a**) and *M. lysodeikticus* (**b**) contacting the materials of interest under visible light irradiation for 10, 20 and 30 min. The data are presented as the mean of three independent measurements, with error bars representing the standard deviation of the mean.

A strong antibacterial effect against *S. aureus* (0% survival cell) after 10 min of visible light irradiation was registered for ZnSe, Lys/ZnSe, ZnSe-TiO_2_ and Lys/ZnSe-TiO_2_ materials (Figure 21a). These results can be correlated with Figure 17a,b,d,e and 18a,b,d,e that clearly indicate the ROS generation over ZnSe-based samples, especially over the unmodified ones (without lysozyme). Still, the formation of hydroxyl radicals over Lys/ZnSe-TiO_2_ and the superoxide anion generation on both Lys/ZnSe and Lys/ZnSe-TiO_2_ samples can explain the high antibacterial activity of the lysozyme-based complexes under light exposure. However, there is a high difference in microbial cell viability when comparing the ZnSe-based samples with the TiO_2_ samples. The results revealed only a slight decrease in the bacteria reduction percentage (13.55%) compared with the control when the bacterium was exposed to the TiO_2_ sample (Figure 21a).

Figure 21b illustrates the antibacterial activity of the investigated samples against *M. lysodeikticus*; a slight increase in the cell survival relative to *S. aureus* is noted. Additionally, by comparison with *S. aureus,* a stronger effect of adding lysozymes can be observed, especially for the TiO_2_ sample. The interaction of lysozyme modified samples with visible light appears to be beneficial for their antibacterial activity. All the above-mentioned results present the overall antibacterial response of the investigated materials, which are dependent on the surface chemistry of the inorganic samples, their capacity to load the lysozyme and activate it and also the specific interaction with bacteria. A previous study, regarding morphological and structural properties of ZnSe and ZnSe-TiO_2_ [17], reported the presence of stilleite as single crystalline phase in a ZnSe sample and stilleite (ZnSe, 85%), zincite (ZnO, 3.3%) and anatase (TiO_2_, 11.9%) as crystalline components of a ZnSe-TiO_2_ sample. Also, the specific surface area for ZnSe and ZnSe-TiO_2_ was found to be 15 m^2^/g and 10. 94 m^2^/g, respectively, proving that the lysozyme loading capacity is slightly higher for ZnSe-TiO_2_ samples than for ZnSe. The buffered powders of ZnSe and ZnSe–TiO_2_ showed no significant differences in the electrokinetic potential (-18.65 mV for ZnSe and -17.95 mV for ZnSe-TiO₂); however, there was a shift toward positive values being obtained for their complexes with lysozyme [17]. Unlike ZnSe-based samples, the present TiO_2_ material displayed a surface area of 130 m^2^/g (Supplementary Information, Figure S3) and a shift to more negative values after lysozyme loading (from −34.9 mV to −42.30 mV, Supplementary Information—Figure S4), which is not favorable to the electrostatic interaction with the negatively charged cell membrane. Despite the higher surface area of TiO_2_, this sample has a lower capacity to load lysozyme than ZnSe and ZnSe-TiO_2_ samples, as resulted from the UV–visible spectra of the collected supernatants from lysozyme loading processes (Supplementary materials—Figure S5). Still, the loaded lysozyme appears to be active against *S. aureus* and *M. lysodeikticus* in dark condition (Figure 19 and Figure 20) and against *M. lysodeikticus* under visible light irradiation (Figure 21b). For identical irradiation experiments, *S. aureus* appears to display a lower resistance to the immobilized lysozyme action (Figure 21a) than *M. lysodeikticus*. By comparing Figure 19, Figure 20 and Figure 21, some differences can be depicted in the reactivity of the investigated samples relative to the same substrate. This can be due to the different experimental conditions: dark and dynamic regime (Figure 19 and Figure 20) versus light irradiation and static regime (Figure 21). For the light assisted tests, the ROS presence was clearly emphasized and proved to be in line with the literature that assesses their antibacterial effect, especially for the hydroxyl radical (•OH), which is known as powerful oxidizing agent. In the present work, Figure 17 proved that •OH is produced mainly by the ZnSe sample and much less by ZnSe-TiO_2_. Figure 18 illustrates the O_2_^•−^ photogeneration by ZnSe and ZnSe-TiO_2_ samples before and after lysozyme immobilization. Generally, the ROS generation over semiconductor-type materials under irradiation in an aerobic medium can be described as follows [30]:

(i) h⁺ + -OH (surface hydroxyl) → •OH
(ii) O_2_ + e^−^ → (O_2_^•−^)_ads_ (on semiconductor surface); (O_2_^•−^)_ads_ + 2H⁺+e^−^ → H_2_O_2_ → 2•OH

In aqueous media, water photolysis can be also a source of •OH:

(iii) H_2_O + hυ → •OH

For our study, to a variable extent, all these mechanisms can to be involved in ROS generation. By correlating these observations with Figure 21, it can be assumed that there is a significant contribution by the photogenerated ROS to the antibacterial activity of ZnSe and ZnSe-TiO_2_ samples against *S. aureus* and *M. lysodeikticus*. The slight antibacterial activity of TiO_2_ is probably due to other mechanisms. On the other hand, the lower antibacterial activity of TiO_2_ against *S. aureus* (Figure 19) than for *M. lysodeikticus* suggests the development of self-defense mechanism(s) of the bacteria. Different structural features of the cell wall and/or a better self-repair capacity of *S. aureus* relative to other bacteria may account for these discrepancies.

Overall, the findings support the favorable impact of lysozyme addition to inorganic carriers on microbial growth inhibition for the investigated systems.

The antibacterial activity of Lys/ZnSe and Lys/ZnSe-TiO_2_ systems is very high and quite similar. In our previous work [17], the enzymatic activity of these hybrid systems relative to a synthetic substrate was slightly higher for Lys/ZnSe-TiO_2_. This comparison indicates presumable differences between the antibacterial activity of lysozyme-derived systems and the enzymatic activity.

## 4. Conclusions

The antibacterial assays involving *S. aureus* and *M. lysodeikticus* showed very good results, both for the bare and most of the lysozyme-modified samples in the dark and when assisted by visible light irradiation.

Bacterial reduction for *S. aureus* (in dark) over unmodified samples was highest in the case of the ZnSe sample. The lysozyme-loaded ZnSe-TiO_2_ and TiO_2_ samples proved to have a better activity than the samples without lysozyme. This was emphasized also by Dark field microscopy characterization that illustrates the direct contact between Lys/ZnSe-TiO_2_ and *S. aureus.*

Unmodified ZnSe-TiO_2_ and ZnSe samples proved to be very efficient against *M. lysodeikticus* (in dark), with the lysozyme loading being beneficial for all samples, especially for TiO_2_.

For *S. aureus*, 100% bacterial reduction was obtained after exposing the ZnSe, ZnSe-TiO_2_, Lys/ZnSe and Lys/ZnSe-TiO_2_ samples to visible light for 10 min. Also, the light exposure proved to be beneficial for enhancing the antibacterial activity of ZnSe, ZnSe-TiO_2_ and TiO_2_ against *M. lysodeikticus* before and after lysozyme loading.

These newly designed light sensitive materials are able to develop lysozyme-based hybrid complexes acting as efficient antibacterial agents under visible light exposure and in the dark.

## Data Availability

Data reported in this study are available on request from the corresponding authors.

## Supplementary Materials

The following supporting information can be downloaded at: https://www.mdpi.com/article/10.3390/antiox12030691/s1, Figure S1: The spectrum of the illumination source with cut off filter λ>420 nm; Figure S2: (a) Qualitative analysis charts of the samples ZnSe-D (top) and ZnSe-Lys (bottom), (b) Qualitative analysis charts of the samples ZnSe-TiO_2_ -D (top) and ZnSe-TiO_2_-Lys (bottom), (c) Qualitative analysis charts of the samples TiO_2_-D (top) and TiO_2_-Lys (bottom); Figure S3: N_2_ adsorption-desorption isotherm and pore size distribution (inset); Figure S4: Zeta potential measurements for TiO_2_ and Lys/TiO_2_; Figure S5: UV-Vis spectra of supernatants containing lysozyme.

## References

[1] Mei L., Zhu S., Liu Y., Yin W., Gu Z., Zhao Y. (2021). An overview of the use of nanozymes in antibacterial applications. Chem. Eng. J..

[2] Sanchez C., Julián B., Belleville P., Popall M. (2005). Applications of hybrid organic–inorganic nanocomposites. J. Mater. Chem..

[3] Cao L. (2005). Immobilised enzymes: Science or art?. Curr. Opin. Chem. Biol..

[4] Anastasescu C., Anastasescu M., Balint I., Zaharescu M. (2019). SiO_2_ based materials for immobilization of enzymes. Nanomaterials—Toxicity, Human Health and Environment.

[5] Jolles P. (1969). Lysozymes: A chapter of molecular biology. Angew. Chem. Int. Ed. Engl..

[6] Sheng L., Wang J., Huang M., Xu Q., Ma M. (2016). The changes of secondary structures and properties of lysozyme along with the egg storage. Int. J. Biol. Macromol..

[7] Calderon C., Abuin E., Lissi E., Montecinos R. (2011). Effect of human serum albumin on the kinetics of 4-methylumbelliferyl-β-D-N-N′-N″ triacetylchitotrioside hydrolysis catalyzed by hen egg white lysozyme. Protein J..

[8] Chifor E., Bordeianu I., Anastasescu C., Calderon-Moreno J.M., Bratan V., Eftemie D.-I., Anastasescu M., Preda S., Plavan G., Pelinescu D. (2022). Bioactive coatings based on nanostructured TiO_2_ modified with noble metal nanoparticles and lysozyme for Ti dental implants. Nanomaterials.

[9] Larsericsdotter H., Oscarsson S., Buijs J. (2001). Thermodynamic analysis of proteins adsorbed on silica particles: Electrostatic effects. J. Colloid Interface Sci..

[10] Shareghi B., Farhadian S., Zamani N., Salavati-Niasari M., Moshtaghi H., Gholamrezaei S. (2015). Investigation the activity and stability of lysozyme on presence of magnetic nanoparticles. J. Ind. Eng. Chem..

[11] Jeon H.-J., Yi S.-C., Oh S.-G. (2003). Preparation and antibacterial effects of Ag–SiO_2_ thin films. Biomaterials.

[12] Guo C., Guo X., Chu W., Jiang N., Li H. (2020). FTIR-ATR study for adsorption of trypsin in aqueous environment on bare and TiO_2_ coated ZnSe surfaces. Chin. Chem. Lett..

[13] Mir I.A., Rawat K., Solanki P.R., Bohidar H.B. (2017). ZnSe core and ZnSe@ZnS core-shell quantum dots as platform for folic acid sensing. J. Nanopart. Res..

[14] Sun C., Gu Y., Wen W., Zhao L. (2018). ZnSe based semiconductor core-shell structures: From preparation to application. Opt. Mater..

[15] Hofmann A., Pettenkofer C. (2011). Surface orientation dependent band alignment for CuInSe_2_-ZnSe-ZnO. Appl. Phys. Lett..

[16] Zhang Z., Fu Y., Yang X., Qu Y., Li Q. (2015). Nanostructured ZnSe anchored on grapheme nanosheets with superior electrochemical properties for lithium ion batteries. Electrochim. Acta.

[17] Anastasescu C., Gifu I.C., Negrila C., Socoteanu R., Atkinson I., Calderon-Moreno J.M., Munteanu C., Plavan G., Strungaru S.A., Cheatham B. (2021). Morpho-structural properties of ZnSe, TiO_2_-ZnSe materials and enzymatic activity of their bioinorganic hybrids with lysozyme. Mat. Sci. Eng. B.

[18] Ibrahim H.R., Matsuzaki T., Aoki T. (2001). Genetic evidence that antibacterial activity of lysozyme is independent ofits catalytic function. FEBS Lett..

[19] Lakshmi Prasanna V., Vijayaraghavan R. (2015). Insight into the mechanism of antibacterial activity of ZnO: Surface, defects mediated reactive oxygen species even in the dark, release of metallic ions and electrostatic interaction of investigated material with cell membrane. Langmuir.

[20] Jiang S., Lin K., Cai M. (2020). ZnO nanomaterials: Current advancements in antibacterial mechanisms and applications. Front. Chem..

[21] Li Y., Zhang W., Niu J., Chen Y. (2012). Mechanism of photogenerated reactive oxygen species and correlation with the antibacterial properties of engineered metal-oxide nanoparticles. ACS Nano.

[22] Kumaravel V., Nair K.M., Mathew S., Bartlett J., Kennedy J.E., Manning H.G., Whelan B.J., Leyland N.S., Pillai S.C. (2021). Antimicrobial TiO_2_ nanocomposite coatings for surfaces, dental and orthopaedic implants. Chem. Eng. J..

[23] Quek J.-A., Lam S.-M., Sin J.-C., Mohamed A.R. (2018). Visible light responsive flower-like ZnO in photocatalytic antibacterial mechanism towards Enterococcus faecalis and Micrococcus luteus. J. Photochem. Photobiol. B Biol..

[24] Neagu S., Anastasescu C., Balint I., Zaharescu M., Ardelean I., Enache M. (2019). The response of Escherichia Coli cells to the action of ZnSe based materials. Rom. J. Mater..

[25] Soheyli E., Sahraei R., Nabiyouni G., Hatamnia A.A., Rostamzad A., Soheyli S. (2018). Aqueous-based synthesis of Cd-free and highly emissive Fe-doped ZnSe(S)/ZnSe(S) core/shell quantum dots with antibacterial activity. J. Colloid Interface Sci..

[26] Anastasescu C., Spataru N., Culita D., Atkinson I., Spataru T., Bratan V., Munteanu C., Anastasescu M., Negrila C., Balint I. (2015). Chemically assembled light harvesting CuO_x_-TiO_2_ p–n heterostructures. Chem. Eng. J..

[27] Whitehouse D.J., Rastogi P.K. (2000). Surface Characterization and Roughness Measurements in Engineering. Photomechanics.

[28] Balint I., Miyazaki A., Aika K.-I. (2001). Alumina dissolution during impregnation with PdCl_4_^2-^ in acid pH range. Chem. Mater..

[29] Moriyama T., Morikawa A. (2017). Sample preparation for X-ray fluorescence analysis. VII. Liquid sample. Rigaku J.—Tech. Artic..

[30] Anastasescu C., Negrila C., Angelescu D.G., Atkinson I., Anastasescu M., Spataru N., Zaharescu M., Balint I. (2018). Distinct and interrelated facets bound to photocatalysis and ROS generation on insulators and semiconductors: Cases of SiO_2_, TiO_2_ and their composite SiO_2_-TiO_2_. Catal. Sci. Technol..

[31] Preda S., Anastasescu C., Balint I., Umek P., Sluban M., Negrila C., Angelescu D.G., Bratan V., Rusu A., Zaharescu M. (2019). Charge separation and ROS generation on tubular sodium titanates exposed to simulated solar light. Appl. Surf. Sci..

