# Supplementary material for: Antibacterial Activity of ZnSe, ZnSe-TiO2 and TiO2 Particles Tailored by Lysozyme Loading and Visible Light Irradiation
Source: Antioxidants (Basel). 2023 Mar 10;12(3):691. doi: 10.3390/antiox12030691 (PMC10045246; doi:10.3390/antiox12030691)
Supplement: Supplementary file 1 [file antioxidants-12-00691-s001.zip › antioxidants-2191908-supplementary.pdf]

Supplementary Information

Antibacterial activity of ZnSe, ZnSe-TiO_2 and TiO_2 particles tailored by lysozyme loading and visible light irradiation

Crina Anastasescu [1], Simona Neagu [2*], Silviu Preda [1], Daniela Culita [1], Mihaela Stancu [2], Cristian Banciu [2], Cornel Munteanu [1], Veronica Bratan [1], Jose Maria Calderon-Moreno [1], Razvan State [1], Mihai Anastasescu [1*], Madalin Enache [2], Ioan Balint [1], Maria Zaharescu [1]

[1] "Ilie Murgulescu" Institute of Physical Chemistry of the Romanian Academy, 202 Spl. Independentei, 060021, Bucharest, Romania; canastasescu@icf.ro (C.A.), munteanuc@icf.ro (C.M.), vbratan@yahoo.com (V.B.), josecalderonmoreno@yahoo.com (J.C.M.), rstate@icf.ro (R.S.), manastasescu@icf.ro (M.A.), ibalint@icf.ro (I.B.), mzaharescu@icf.ro (M.Z.)
[2] Institute of Biology of the Romanian Academy, 296 Splaiul Independenţei, Bucharest 060031, Romania; si-mona.neagu@ibiol.ro (S.N.), mihaela.stancu@ibiol.ro (M.S.), madalin.enache@ibiol.ro (M.E.)
Correspondence: manastasescu@icf.ro (M.A.); simona.neagu@ibiol.ro (S.N.); Tel.: +40-21-3167912 (M.A.); +40-21-2219202 (S.N.)

I. **Spectrum of the illumination source**

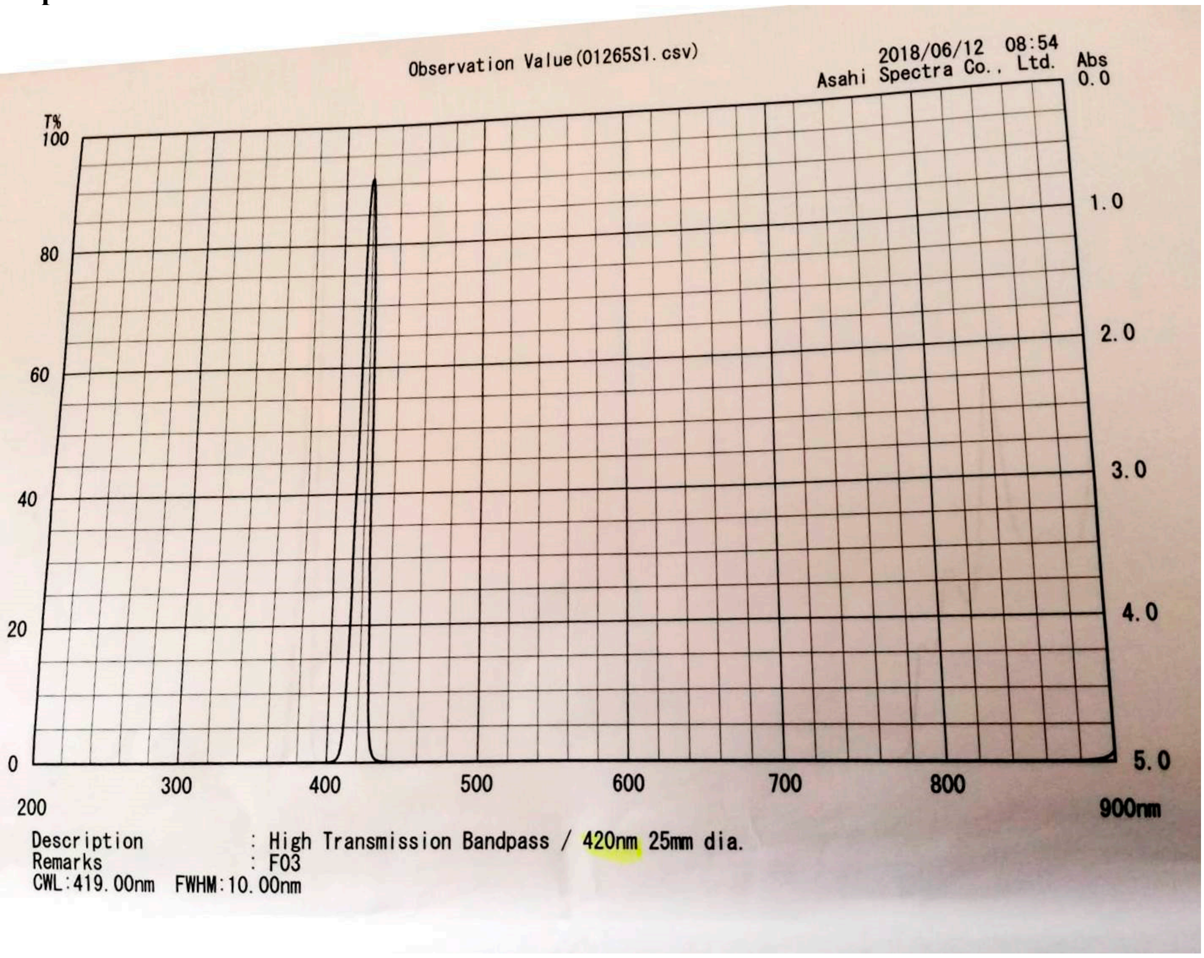

Figure S1. The spectrum of the illumination source with cut off filter λ>420 nm

II. ***X−ray fluorescence (XRF) characterization*** *for ion releasing measurements*

X−ray fluorescence (XRF) was used for elemental analysis. The measurements were performed using a Rigaku ZSX Primus II spectrometer (Rigaku Corp., Tokyo, Japan), equipped with 4.0 kW X−ray Rh tube. EZ−scan combined with Rigaku SQX fundamental parameters software (standard less) was used for data analysis.

For the liquid samples, the droplet method was used. According to Moriyama et al.[29], droplet method (or filter paper method) is used for analysis of dried droplet solution on special filter paper

100 µl of sample solution is droped onto the filter paper. The solution was dried at room temperature (below 60 °C in order to avoid the risk of melting of wax that prevents expansion of sample solution and warping of filter paper). ZnSe, TiO_2, ZnO nanoparticles are hindered to pass through membrane from aqueous suspension in the liquid sample collected for analysis, only ions are presumable to be present [28]

Six samples were measured by the droplet method and the qualitative analysis charts of the heavy elements (that contains our area of interest) are presented below.

In the Figure S2 (a), the ZnSe and Lys/ZnSe samples referred to as ZnSe-D and ZnSe-Lys are shown. Zinc was detected only in the sample ZnSe-D. Since the filter paper is very thin that X-rays is transmitted easily, impurity X-rays (Fe, Ni) originated from sample holder or sample support are detected.

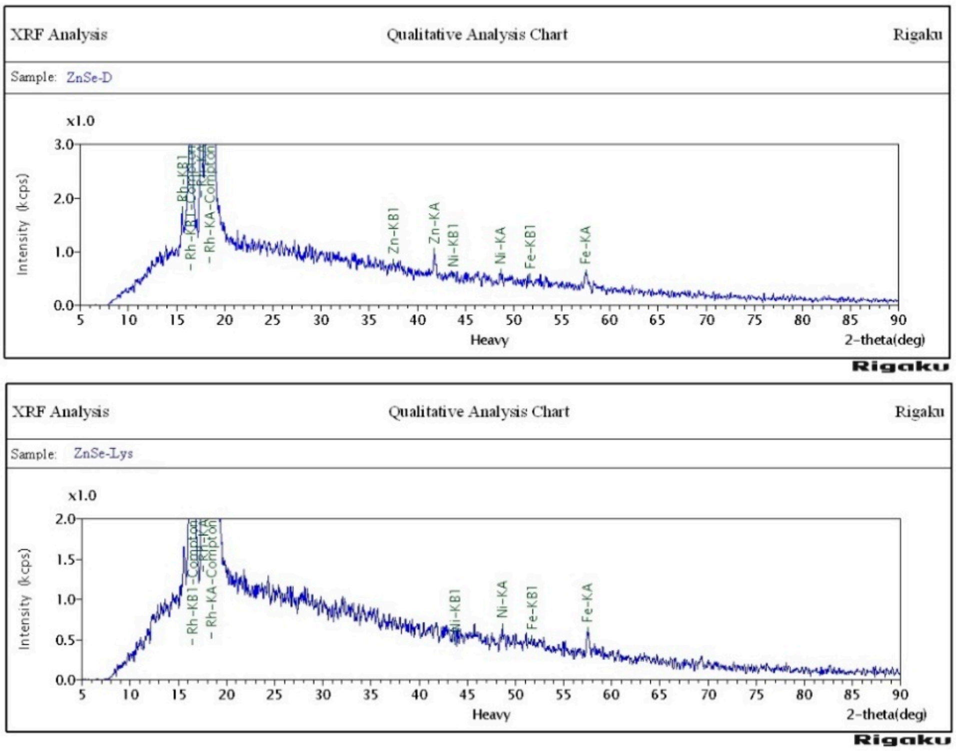

Figure S2 (a). Qualitative analysis charts of the samples ZnSe-D (top) and ZnSe-Lys (bottom)

In the Figure S2 (b), the samples ZnSe-TiO_2 and Lys/ZnSe-TiO_2 referred to as ZnSe-TiO_2-D and ZnSe-TiO_2-Lys are shown.

In the Figure S2 (c) the samples TiO_2 and Lys/TiO_2 referred to as TiO_2-D and TiO_2-Lys are exhibited, respectively. According to Figure S2 (b) and (c), no zinc, selenium or titanium ions were detected for the samples ZnSe-TiO_2, Lys/ ZnSe-TiO_2, TiO_2, Lys/TiO_2.

The quantity of zinc deposited on the filter paper after drying was calculated for the sample ZnSe-D. After extracting the filter paper data (cellulose), a quantity of 0.3 µg/cm^2 was obtained.

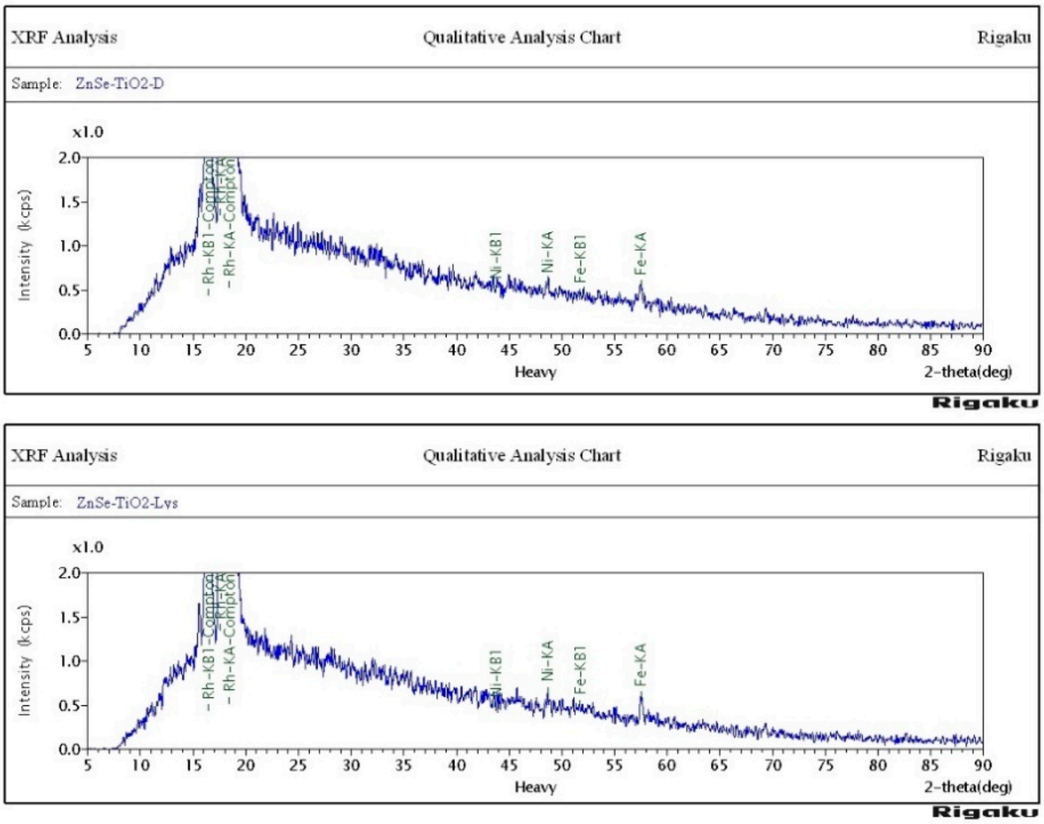

Figure S1 (b) Qualitative analysis charts of the samples ZnSe-TiO_2 -D (top) and ZnSe-TiO_2-Lys (bottom)

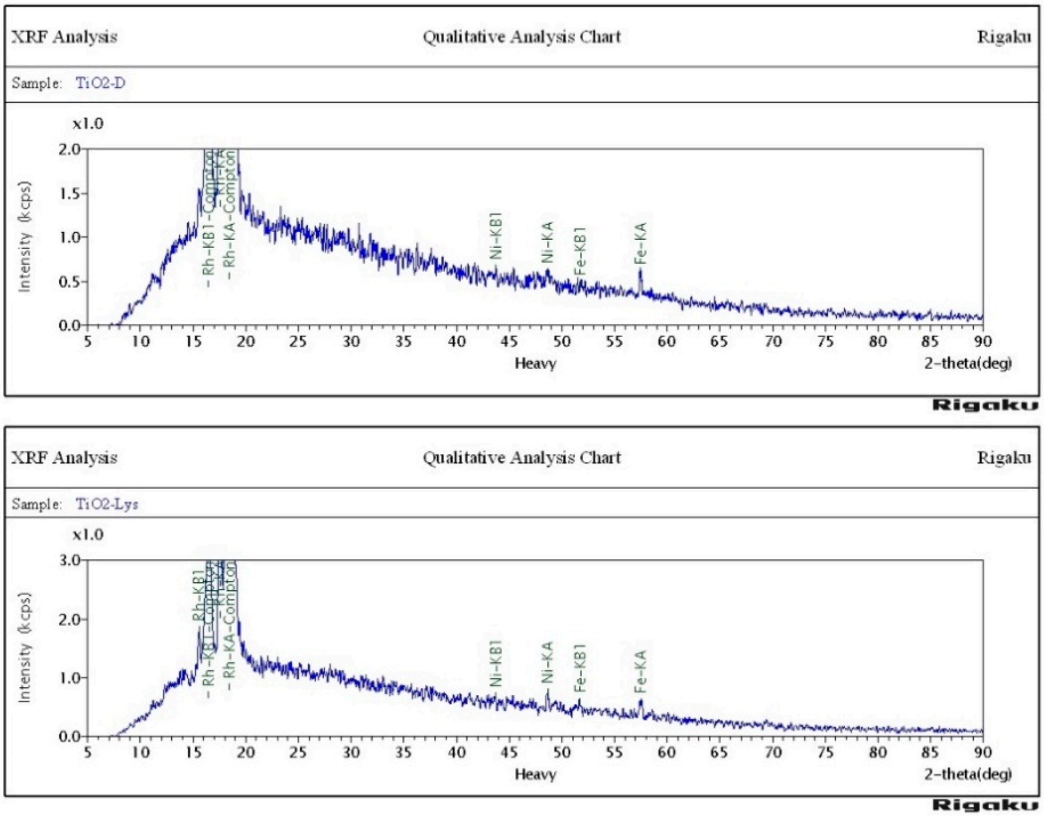

Figure S2 (c). Qualitative analysis charts of the samples TiO_2-D (top) and TiO_2-Lys (bottom)

III.**Nitrogen adsorption-desorption** measurements for TiO_2 sample were performed by using Micromeritics ASAP 2020 Plus 2.00.

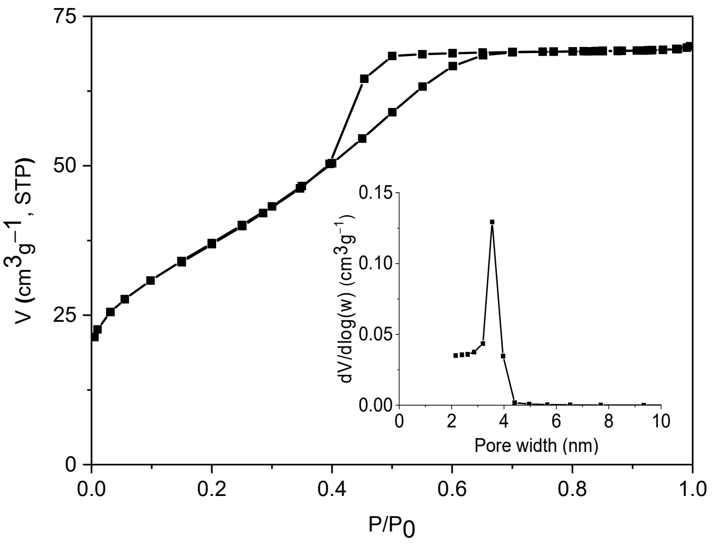

Figure S3 N_2 adsorption-desorption isotherm and pore size distribution (inset)

Based on the adsorption data and using Brunauer-Emmett-Teller (BET) equation, specific surface area (S_{BET}) was found to be 135.3064 m²/g
By using desorption branch data and Barrett–Joyner–Halenda (BJH) method, the pore size distribution curve was obtained .

IV. Zeta potential measurements for TiO_2 and Lys/TiO_2 in aqueous media performed with Delsa Nano C particle analyzer (Backman Coulter Brea, CA, USA).

Distribution Graph S/N :

User	: Common	Group	:	Repetition : 1/1
Date	: 16/02/2023	File Name	: TiO2_20230216_111642	
Time	: 11:16:42	Sample Information	:	
SOP Name	: Rompharm5			Security : No Security

Version 2.31 /

Mobility Distribution

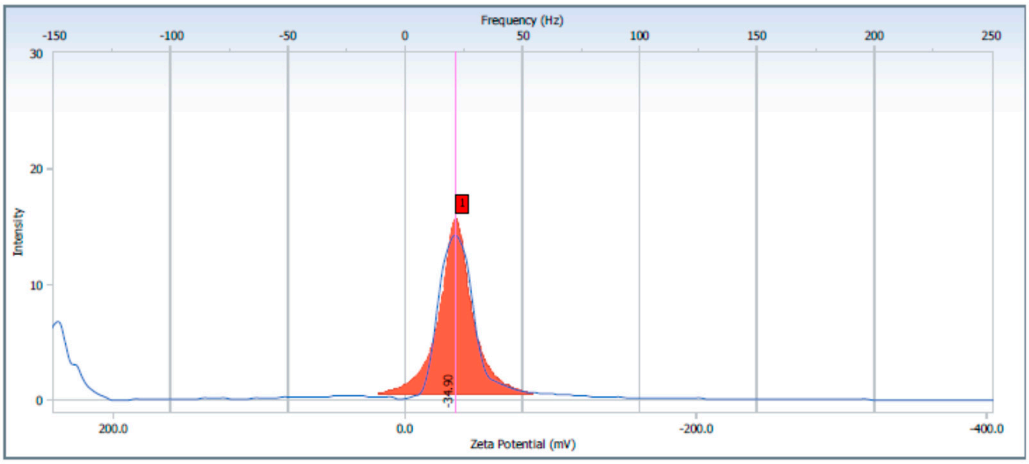

Measurement Results

Zeta Potential : -34.90 (mV) Doppler shift : 21.67 (Hz)

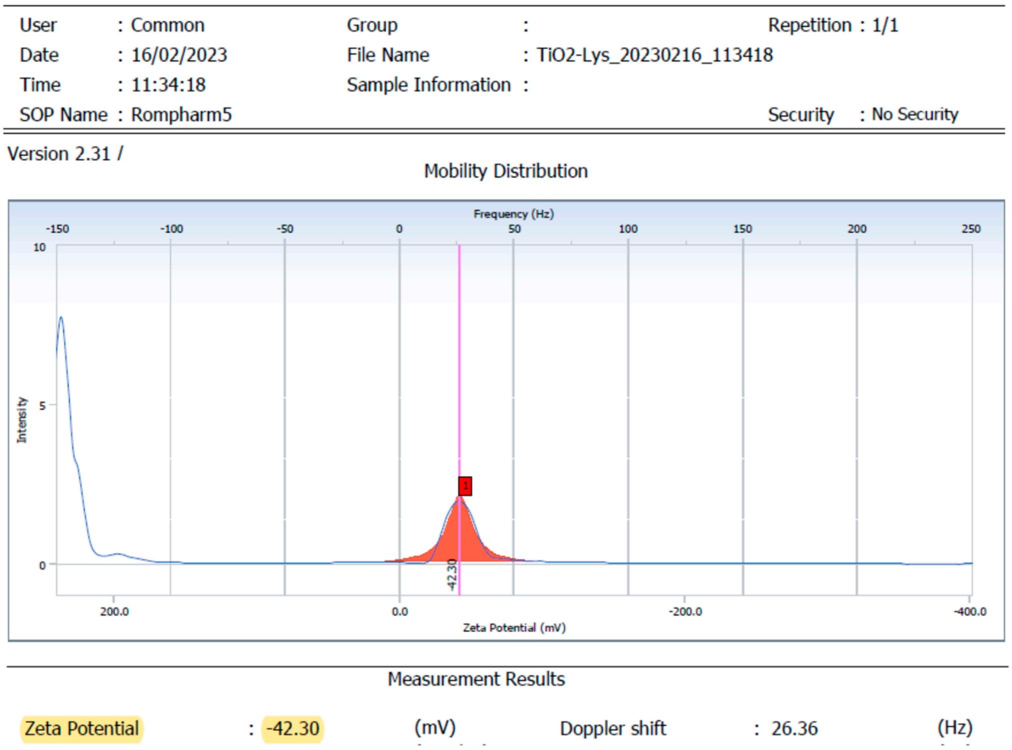

Figure S4 Zeta potential measurements for TiO_2 and Lys/TiO_2

V. UV-Vis absorption spectra for liquid samples (removed supernatants from lysozyme loading on ZnSe, ZnSe-TiO_2 and TiO_2) were performed with an Analytik Jena Specord 200 Plus spectrophotometer.

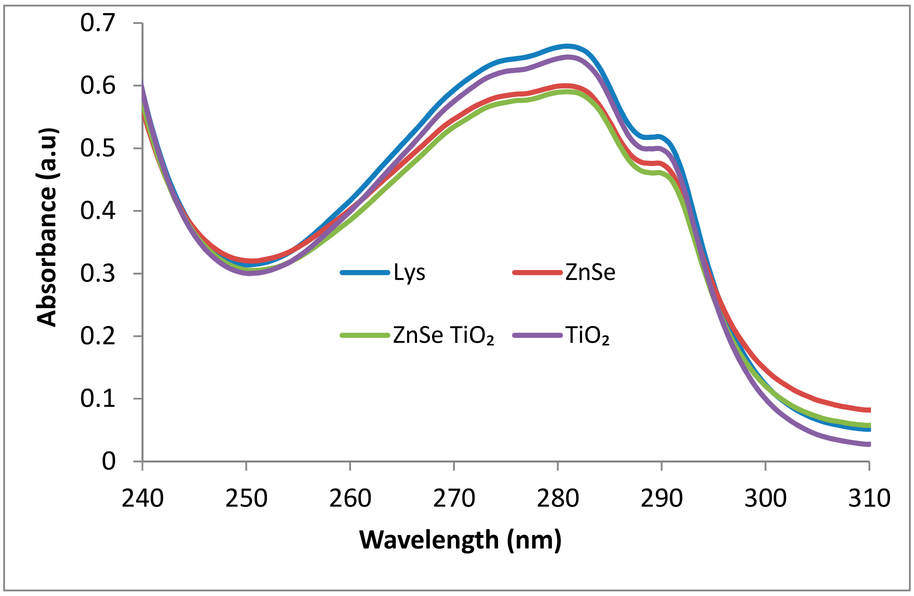

Figure S5. UV-Vis spectra of supernatants containing lysozyme

The sequence of lysozyme concentration in the investigated samples was, the sample ZnSe –TiO_2 and ZnSe being almost similar:

ZnSe-TiO_2< ZnSe<TiO_2< Lys

Accordingly, the amount of lysozyme loaded on the inorganic sample is increasing as follows: TiO_2< ZnSe< ZnSe-TiO_2
